# Genetic and Epigenetic Changes in Oilseed Rape (*Brassica napus* L.) Extracted from Intergeneric Allopolyploid and Additions with *Orychophragmus*

**DOI:** 10.3389/fpls.2016.00438

**Published:** 2016-04-12

**Authors:** Mayank Gautam, Yanwei Dang, Xianhong Ge, Yujiao Shao, Zaiyun Li

**Affiliations:** ^1^National Key Laboratory of Crop Genetic Improvement, National Center of Oil Crop Improvement (Wuhan), College of Plant Science and Technology, Huazhong Agricultural UniversityWuhan, China; ^2^College of Chemistry and Life Science, Hubei University of EducationWuhan, China

**Keywords:** allopolyploidization, monosomic additions, transposons, cytosine methylation, *Brassica napus*

## Abstract

Allopolyploidization with the merger of the genomes from different species has been shown to be associated with genetic and epigenetic changes. But the maintenance of such alterations related to one parental species after the genome is extracted from the allopolyploid remains to be detected. In this study, the genome of *Brassica napus* L. (2*n* = 38, genomes AACC) was extracted from its intergeneric allohexaploid (2*n* = 62, genomes AACCOO) with another crucifer *Orychophragmus violaceus* (2*n* = 24, genome OO), by backcrossing and development of alien addition lines. *B. napus*-type plants identified in the self-pollinated progenies of nine monosomic additions were analyzed by the methods of amplified fragment length polymorphism, sequence-specific amplified polymorphism, and methylation-sensitive amplified polymorphism. They showed modifications to certain extents in genomic components (loss and gain of DNA segments and transposons, introgression of alien DNA segments) and DNA methylation, compared with *B. napus* donor. The significant differences in the changes between the *B. napus* types extracted from these additions likely resulted from the different effects of individual alien chromosomes. Particularly, the additions which harbored the *O. violaceus* chromosome carrying dominant rRNA genes over those of *B. napus* tended to result in the development of plants which showed fewer changes, suggesting a role of the expression levels of alien rRNA genes in genomic stability. These results provided new cues for the genetic alterations in one parental genome that are maintained even after the genome becomes independent.

## Introduction

Allopolyploidization with combination of two or more divergent genomes through interspecific hybridizations is a shaping force in the plant speciation (Ramsey and Schemske, 1998), as it lead to immediate evolutionary advantage, such as adaptation to novel niches and changing environment (Parisod et al., 2010b). At chromosomal and genomic levels, polyploidy can lead to gene redundancy (Adams and Wendel, 2005; Moore and Purugganan, 2005), translocation and transposition (interchromosomal exchanges), unequal crossing over, transposon-mediated event, mutation, sequence deletion and insertion (illegitimate recombination) (Chen, 2007; Doyle et al., 2008). Studies on serial resynthesized polyploids (e.g., *Arabidopsis, Brassica, Gossypium, Nicotiana, Triticum*, and *Triticale*) and natural polyploid species have revealed genomic changes in nascent polyploid taxa, including deletion events, gene conversion events, rDNA loci changes, transposon activation, chromosomal rearrangements, and epigenetic phenomena (Comai, 2005; Chen, 2007; Doyle et al., 2008). Also, allopolyploidy is considered to play a major role in gene expression especially non-additive expression of homoeologous genes (Chen, 2007; Li et al., 2014).

In contrast to allopolyploidy, aneuploidy refers to karyotypic abnormalities with the gain or loss of one or more chromosomes from the normal number (2*n*) in the cells, which disrupts the balanced complement and has detrimental effects on various aspects of growth and developments in all organisms studied (Siegel and Amon, 2012). The abnormalities in phenotype and development are attributable to the genome-wide gene expression perturbation revealed in plants and animals (Letourneau et al., 2014; Zhu et al., 2015). Aneuploidy may increase genomic instability (increased rates of chromosome mis-segregation, mitotic recombination, mutation, increased DNA damage), resulting in the reduced cell fitness (Janssen et al., 2011; Crasta et al., 2012). However, plants appear to be remarkably resistant to the adverse effects of ploidy and show high viability (Makarevitch and Harris, 2010); aneuploidy may play a role in both speciation and genome evolution (Ramsey and Schemske, 2002).

Monosomic alien addition lines are another type of aneuploids that have one chromosome added to their genome from an alien species. A complete set of MAALs, each line carrying a chromosome of an alien species can be developed by recurrent backcrossing of the natural or synthesized allopolyploids with one of the parental species, which results in the dissection of the genome of the donor species. The complete set of MAALs is widely used for studying the genome structure, genetic mapping and breeding bridge to transfer target genes and traits from the alien chromosome to the recipient genome (Zhao et al., 2008; Prakash et al., 2009; Geleta et al., 2012; Ding et al., 2013; Kang et al., 2014). But how the individual alien chromosomes impact the host genome remain to be elucidated. The extent of the genetic and the epigenetic changes in the genome of one parent effected by allopolyploidization/aneuploidization can be determined by comparing the parental species among the progenies resulting from self-pollination of the MAALs.

The globally important oilseed rape (*Brassica napus* L.) is a young allotetraploid species (2*n* = 4x = 38, genomes AACC); it originated ∼7500 years ago from natural interspecific hybridization between *B*. *rapa* L. (2*n* = 20, genome AA) and *B*. *oleracea* L. (2*n* = 18, genome CC) (Chalhoub et al., 2014). *B*. *napus* has been one of the model system for studying genetic and phenotypic changes associated with allopolyploid formation at genomic, transcriptomic and proteomic levels (Song et al., 1995; Albertin et al., 2006; Lukens et al., 2006; Gaeta et al., 2007, 2009; Xu et al., 2009; Ksiazczyk et al., 2011; Xiong et al., 2011; Zhou et al., 2011; Cui et al., 2012, 2013), together with another model system *Arabidopsis suecica* (Pontes et al., 2004; Chen, 2007). A set of nine MAALs of *B. napus* with one of the different chromosome from another crucifer *Orychophragmus violaceus* (L.) O. E. Schulz (2*n* = 24, genome OO) have been obtained and characterized for their phenotype and cytology (Ding et al., 2013). These MAALs were developed by producing the intergeneric somatic hybrid/allohexaploid with the sum of the chromosomes from these two species (2*n* = 6x = 62, genomes AACCOO) and then recurrent backcrossing of the allohexaploid with *B. napus* (Zhao et al., 2008; Ding et al., 2013). These lines were distinguishable from each other, because they expressed the phenotypes and chromosome markers specific for *O. violaceus*. In particular, the rDNA loci on three *O. violaceus* chromosomes were active and showed variable degrees of expression dominance over those of *B. napus*, i.e., the epigenetic phenomenon of nucleolar dominance (ND) (Chen, 2007). Some phenotypic deviations from the original *B. napus* genotype were presented by the self-pollinated *B. napus*-type progenies (2*n* = 38) from these MAALs, which were likely attributable to the alien genetic introgression from *O. violaceus*, to the genetic changes during the process of intergeneric hybridization/allopolyploidization/aneuploidization. In this study, the *B. napus*-type progenies derived from nine MAALs were investigated and compared by methods of AFLP, SSAP and MSAP, to quantify the genomic changes, retrotransposon mobilization and alterations in cytosine methylation. These results have provided novel insights into the genetic and epigenetic modifications that have persisted even after the genome was restituted from the allopolyploids.

## Materials and Methods

### Plant Materials

**FIGURE 1:**
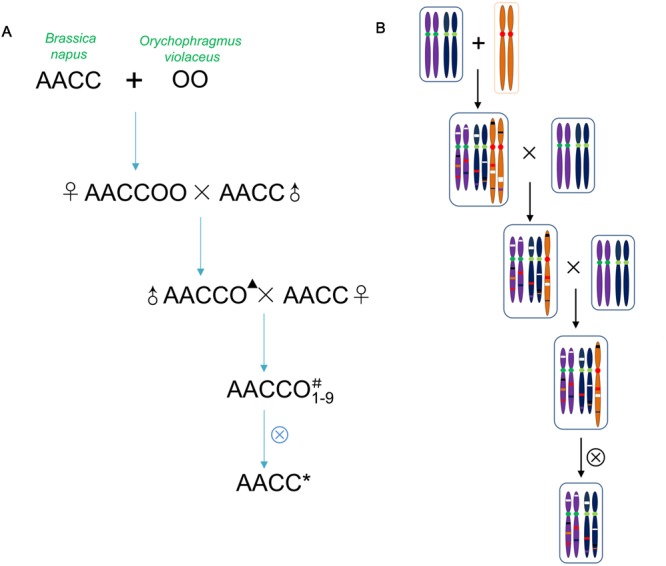
**Extraction procedure of *Brassica napus*-type progeny from the allohexaploid **(A)** and presence of the genetic changes on the chromosomes **(B)**.** One pair of chromosomes for each genome was shown. The genetic changes include exchanged segments between genomes of two parents. The changes represented by different colors, lost bands (white), new bands (red) and the epigenetic changes (black). ▴Represented the haploid genome of *O*. *violaceus*. ^#^Represented one chromosome of *O*. *violaceus*. ^∗^Represented the extracted *B. napus* with the genetic and the epigenetic changes.

The intergeneric somatic hybrid No. 101 (2*n* = 62, genomes AACCOO) of *Brassica napus* L. cv. Huashuang No. 3 (2*n* = 38, genomes AACC) and *O. violaceus* (L.) O. E. Schulz (2*n* = 24, genome OO) was pollinated with Huashuang No. 3. The backcrossed progeny produced (2*n* = 50, genomes AACCO) was female sterile but male fertile (Zhao et al., 2008). Among the further backcrossed progenies (Huashuang No. 3 as the female parent), nine different monosomic (i.e., 2*n* = 39, AACC + 1O; where 1O represented a single *O*. *violaceus* chromosome) or disomic additional lines (i.e., 2*n* = 40, AACC + 2O; where 2O represented a pair of *O*. *violaceus* chromosomes) were established (designated as M1–M9) (**Figure 1**). Eight of them reported previously were distinguishable from each other on the basis of their phenotype and cytology (Ding et al., 2013). Five plants having the same number of chromosomes as *B. napus* (2*n* = 38) among the selfed progenies from each of these additional lines except the female sterile one M7 were identified to study the genetic changes induced during the process of allopolyploidization and aneuploidization, in comparison with parental *B. napus*. Because M7 was female sterile but male fertile, *B. napus*-type progenies were produced by pollinating the parental Huashuang No. 3. The selfed seeds of these *B. napus*-type plants were used to obtain the plants for DNA extraction, for the investigations of phenotype, fertility and seed quality. The parental Huashuang No. 3 was also selfed in each generation in parallel with the somatic hybrids and progenies.

### Cytology and Pollen Fertility

The chromosome complement of 45 individuals of the *B. napus*-type progeny (2*n* = 38) raised from nine selfed addition lines was determined by chromosome counting of the ovaries from young flower buds and by the meiotic pairing and segregation in pollen mother cells (PMCs). The immature ovaries of young flower buds were treated with 2 mM 8-hydroxyquinoline for 3–4 h at room temperature and then fixed in acetic acid: ethanol 1:3 (v/v) and used for counting the number of chromosomes according to the procedure of Li et al. (1995). For studying meiotic analysis in PMCs, young flower buds were fixed and stored at -20°C. At anthesis, pollen samples were collected closed to noon time on sunny days and stained with 1% acetic carmine to score pollen stainability as an indicator of pollen fertility.

### Amplified Fragment Length Polymorphisms

The AFLP procedure was performed with parent plants and with progenies resulting from selfing, according to the protocol of Vos et al. (1995). Genomic DNA was extracted from young fresh leaves by the cetyltrimethyl-ammonium bromide procedure (Kidwell and Osborn, 1992). In total, 32 pairs of AFLP primers (Supplementary Table S2) were randomly selected and used for AFLP fingerprint analysis. Each AFLP gel was run twice. The upper part and lower part of the gel, where resolution was not satisfactory, was not used for band scoring. Only high-resolution, middle-part of the gel, which was reproducible in all cases were used for scoring.

### Sequence-Specific Amplification Polymorphisms

In the present investigation, the SSAP strategy was mainly used following the protocol of Pouilly et al. (2008) which was based on previously described protocols of Waugh et al. (1997) and Ellis et al. (1998) with some modifications (Zou et al., 2011; Zhang et al., 2013) to design the primer sequences (Supplementary Table S3). The endonuclease enzyme *Eco*RI was used to restrict genomic DNA, which was employed following ligation to adaptors, as the template for a two-step SSAP reaction including pre-amplification and selective amplification. We surveyed three contrasting *Brassica* transposons, *Athila*-like retrotransposon, *BraSto*, and *Bot1*.

### Methylation-Sensitive Amplification Polymorphisms

The methylation-sensitive amplified fragment length polymorphism (MSAP) protocol (Xiong et al., 1999; Xu et al., 2000) was used as described by Zhang et al. (2013). The 23 pairs of randomly selected primers (Supplementary Table S4) were used to detect the DNA methylation alteration patterns; only the clear and reproducible bands were scored. MSAP involved the use of two isoschizomers, *Hpa*II and *Msp*I which can recognize the similar CCGG sites that have different sensitivity to the methylation states of the cytosine. *Hpa*II is inactive if one or both strands of cytosine are fully methylated, but it cleaves hemi-methylated sequences (i.e., if only a single DNA strand is methylated), or neither strand is methylated; *Msp*I digests methylation of double-stranded DNA, or non-methylated sequences. Based on the presence (+) or absence (-) of a band in the *Eco*RI/*Hpa*II-digested (H) or *Eco*RI/*Msp*I-digested (M) genomic DNA, the methylation status of the specific loci was decided.

### Fatty Acid Analyses

Seed oil was extracted and its composition was analyzed using gas chromatography (HP 6890, Germany). The content of fatty acid and glucosinolate were estimated using near infrared reflectance spectroscopy (NIRS) (Vector 22/N, Bruker, Germany).

### Statistical Analyses

Two-by-two chi-square contingency tests were used to test the significance of the genetic and the epigenetic changes among the *B*. *napus*-type progenies and the Pearson correlations were performed among the observed changes.

## Results

### Phenotype and Fertility of *B. napus* Plants Extracted from Alien Additions

Among the self-pollinated progenies of each of nine *B. napus*–*O. violaceus* addition lines (M1–M9) (**Figure 1**), five *B. napus*-type plants (2*n* = 38) were identified for further study by cytological investigations including, chromosome counting in the somatic cells and normal meiotic pairing (19 bivalents) and 19:19 segregation in PMCs. All these 45 plants showed high pollen fertility (90.5–96.8% stainability) and good seed set after selfing, while the parental *B. napus* cv. Huashuang No. 3 showed expectedly normal pollen fertility and seed-setting. They showed the phenotype similar to Huashuang No. 3, except for minor differences in leaf shape and color. Nine lines derived from the 45 plants largely maintained the phenotype of Huashuang No. 3, but some differences in shape and color of leaves, flowering time and fertility were discernible between the lines from the same or different additions. The individuals from M9 showed delayed flowering and low seed set. The selfed seeds of these nine lines exhibited low content of erucic acid (0.47–3.31%) and variable contents of glucosinolates (11.82–65.51 μmol/g ∼30 μmol/g), as in Huashuang No. 3 which also showed low contents of erucic acid (∼1%) and glucosinolates (∼30 μmol/g) in seeds.

### AFLP Analysis of Genomic Changes in the Extracted *B. napus* Plants

The genomic components of these 45 *B*. *napus*-type individuals were analyzed using 32 primer combinations of EA (*Eco*RI) and MC (*Mse*I). In all, 5,534 polymorphic bands were scored; this comprised 3,537 (63.9%) bands lost in the parent *B. napus* but present in the progenies, 937 (16.9%) new bands unobserved in both parent, and 1,060 (19.2%) bands specific to *O. violaceus*. The number of bands lost ranged from 13 in line M5 to 256 in line M6, that of the novel bands ranged from 4 in line M8 to 31 in line M3, and that of the *O*. *violaceus*-specific bands was from 6 in line M6 and M9 to 33 in line M3. The average of the lost bands (78) was much higher than the averages of the novel bands (21) and *O*. *violaceus*-specific bands (23). Among the five plants from the same addition line, the number and percentage of each kind of polymorphic band varied only narrowly for most additions, except the line M6 and M8; in M6 the number of the bands that were lost ranged from 60 to 256, and that of the *O*. *violaceus*-specific bands from 6 to 25. In M8 the number of the novel bands ranged from 4 to 24. These data indicated that the lost bands varied much more widely among the plants.

The average rates of all the three kinds of bands for the five plants within each addition showed no significant differences for M2, M3, M4, M5 (Supplementary Table S1). Compared to the M2–M5, the average rate of the lost band was significantly higher than the average rate of the new bands as well as that of the *O*. *violaceus*-specific bands in lines M1, M6, M8, and only significantly higher than that of new bands but not than that of *O*. *violaceus*-specific bands in line M7. Additionally, the average rate of the lost band was significantly higher than the average rate of the new bands as well as that of the *O*. *violaceus*-specific bands in lines M1, M6, M8, and M9, this possibly contributed to the higher total rates (2.58–3.40%) in these four lines. Unlike these four lines, the other four lines M2, M3, M4, and M7 were only marginally higher. Line M5 was an exception; the average rate of the lost band was lower than the average rate of other two types of bands (Supplementary Table S1). The plants from each addition presented the higher rate of lost band but lower rates of both new and specific bands (except for M5) than the others (1.46–1.76).

**FIGURE 2:**
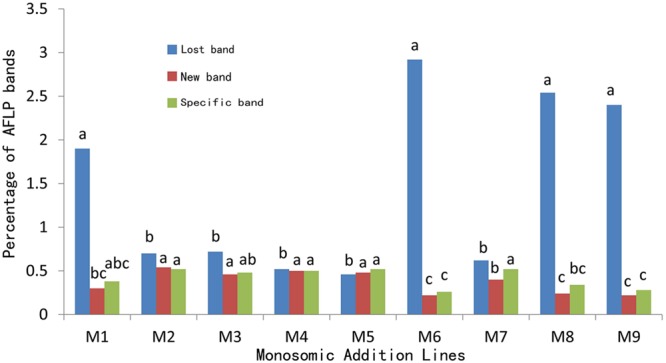
**Average percentages of AFLP bands in the extracted *B. napus*-type plants.** The percentages represented by the bars of the same color marked with identical letter (s) are not significant (*p* < 0.05).

For the comparison of the average rates of lost bands among plants from different additions (**Figure 2**), the percentage for line M6 (2.92) was highest but showed no significant differences as compared to M1 (1.90), M8 (2.54), and M9 (2.40); however, these percentages were significantly higher than those of the remaining additions (0.46 for M5 to 0.72 for M3). For the comparison of novel bands, the rates for M2–M5, and M7 were comparable but were higher than those of M6, M8, and M9. As to the specific bands, the rates for M2–M5, and M7 were similar but significantly higher than those of M6 and M9.

### SSAP Analysis of Transposon Variations in the Extracted *B. napus* Plants

Sequence-specific amplified polymorphism markers were used to detect the variations in three contrasting transposable elements, namely *Athila*-like retrotransposons, *Bot1*, and *BraSto* in the 45 *B. napus*-type individuals from the nine addition lines (**Table 1**). SSAP polymorphism appeared as lost bands as well as new bands, but the percent of the new bands was rather low; in fact no new band appeared for *Bot1* in any progenies. Like the AFLP bands, the number of SSAP lost bands varied not only among the lines but also among the five individuals within a given lines. We found that the number of lost bands among the 45 plants varied from 1 to 46 for *Athila*-like, from 1 to 29 for *Bot1*, and from 1 to 12 for *BraSto*, and averaged 16, 7.5, 4.6, respectively. The widest range of the lost bands among the five plants in the same additions were 4–35 for *Athila*-like and 1–10 for *BraSto*, both in the line M8, and 7–29 for *Bot1* in the line M6.

**Table 1:** Statistical analysis of the SSAP bands in *Brassica napus*-type progenies derived from nine additions^∗^.

Transposon type	*Athila*-like		*Bot1*		*BraSto*		Total	
Line	Lost band	New band	Lost band	New band	Lost band	New band	Lost band	New band
M1	↑^B^2.08^bdc#^	0	^A^3.48^b^	0	^B^2.12^ab^	0.16^c^	7.68	0.16
M2	^B^2.86^abc^	^B^0.06^a^	^C^2.26^bc^	0	^A^3.54^a^	^A^0.64^ab^	8.66	0.70
M3	^A^3.14^ab^	^B^0.04^a^	^A^2.48^bc^	0	^A^2.80^ab^	^A^0.40^abc^	8.42	0.44
M4	^A^0.80^d^	^B^0.06^a^	^A^0.78^c^	0	^A^0.54^d^	^A^0.80^a^	2.14	0.86
M5	^A^2.10^bdc^	^B^0.02^a^	^A^1.96^bc^	0	^A^1.50^bcd^	^A^0.72^ab^	5.56	0.74
M6	^AB^4.20^a^	^B^0.06^a^	^A^5.78^a^	0	^B^2.70^ab^	^A^0.32^bc^	12.68	0.38
M7	^A^1.12^cd^	^B^0.06^a^	^A^0.36^c^	0	^A^0.68^cd^	^A^0.56^abc^	2.16	0.62
M8	^A^2.70^abc^	^B^0.02^a^	^A^2.60^bc^	0	^A^1.58^bcd^	^A^0.40^abc^	6.88	0.42
M9	^A^0.66^d^	0	^A^0.36^c^	0	^A^0.76^cd^	0.48^abc^	1.78	0.48
Mean	2.18	0.04	2.23	0	1.80	0.50		

*^∗^All values are in percentage. ↑Values for a given kind of band across a progeny line marked with an identical capital letter (s) are not significant (*p* < 0.05). # Values in each column marked with an identical letter (s) are not significant (*p* < 0.05).*

The highest rate of the lost bands of the *Athila*-like transposons in M6 (4.2) was insignificant with the reference to the percentages in M2 (2.86), M3 (3.14), and M8 (2.70), but significantly higher than those of the remaining five lines, M1 (2.08), M4 (0.80), M5 (2.10), M7 (1.12), and M9 (0.66) (**Table 1**). The rates of the new bands of the *Athila*-like transposons were quite low (0–0.02) and had no obvious differences. Of the lost bands of *Bot1*, the percentage for M6 (5.78) was significantly higher than that of all other lines. The percentages for M1 (3.48), M2 (2.26), M3 (2.48), M5 (1.96), and M8 (2.60) were comparable. Likewise, only the percentage for M1 was significantly higher than those of M4 (0.78), M6 (0.36), and M9 (0.36). Of the lost bands of *BraSto*, although the percentage for the M2 (3.54) was the highest and it was not significantly higher than the percentages for the line M1 (2.12), M3 (2.80), and M6 (2.70), but it was significantly higher than the percentages for the remaining five lines, M4 (0.54), M5 (1.5), M7 (0.68), M8 (1.58), and M9 (0.76). When the new bands of *BraSto* were considered, the rates of the new bands of *BraSto* were low (0.16–0.80) and only the percentage for M4 (0.80) was significantly higher than those of M1 (0.16) and M6 (0.32). For the total rates of absent bands of these three transposons (1.78–12.68), M6 (12.68) was much higher than all others, and M1, M2, M3, M5, and M8 were ranged from 5.56 to 8.66 which was higher than M4, M7, and M9 having range between 1.78 and 2.14. The total rates of new bands of these three transposons were still low (0.16–0.86).

For the comparisons of the frequencies of the absent bands between three transposons within each addition (**Table 1**), the rate for *Bot1* (3.48) in line M1 was significantly higher than those of *Athila*-like (2.08) and *BraSto* (2.12). Within line M2, the rate of *BraSto* (3.54) was significantly higher than that of *Athila*-like (2.86) which was also significantly higher than that of *Bot1* (2.26). For M6, the rate of *Bot1* (5.78) was significantly higher than that of *BraSto* (2.70) but not than that of *Athila*-like (4.20). Within the remaining six additions, there were no significant differences among three rates. As to the rates of new bands which occurred both for *Athila*-like and *BraSto* within seven additions, the rates of *BraSto* were significantly higher than those of *Athila*-like transposons.

### MSAP Analysis of DNA Methylation Alterations in the Extracted *B. napus* Plants

As already mentioned, the MSAP protocol described by Zhang et al. (2013) was followed; 23 pairs of primers were used to detect the DNA methylation alteration patterns. The MSAP loci in these *B. napus*-type progenies were grouped into fifteen patterns and compared with those in the parent *B*. *napus* (**Table 2**). Fifteen patterns of methylation were recognized; three pattern namely ++/++, ++/-- and --/++ were excluded from the MSAP analysis as it was not possible to decide whether the loci were methylated (++/++) or they resulted from the methylation changes, or sequence variation (++/-- and --/++). The remaining 12 patterns were either monomorphic or polymorphic and were grouped into A, B, C, D types (**Table 3**); monomorphic pattern indicates similar pattern of methylation in the parent as well as the progenies in either *Hpa*II (H) or *Msp*I (M) lane. Three types of DNA methylation—hypermethylation (type A), hypomethylation (type B), and variable pattern of methylation (type C) were observed. Type A referred to the hypermethylation state in the parent *B. napus*, and Type B to the hypomethylation state in the progenies. Types A and B presented four subtypes each—A_1_ to A_4_ and B_1_ to B_4_. A_1_ was the *de novo* methylation in the parent *B*. *napus*, A_2_ was the *de novo* methylation in the progeny lines (in either *Hpa*II or *Msp*I lane), A_3_ was hypermethylation in the parent (in either *Hpa*II or *Msp*I lane), and A_4_ was the hypermethylation band in the progeny. The rates of Type A within additions were much higher than those of Type B, except for the reverse situation in M2. Therefore, the methylation changes were mainly observed in type A and type B (**Table 3**). Type C represented varied patterns of methylation; these patterns showed no difference in DNA methylation between the parent and the progenies. Type D showed three subtypes—D_1_ (no methylation), D_2_ (hemi-methylation), and D_3_ (hemi-methylation). The percentage of Type D varied from 41.20 to 66.0 among the 45 plants and showed an average of 53.08–62.42 among additions (**Table 3**); this suggested that the progeny lines inherited most of the methylation patterns from the parent *B*. *napus*. That the percentage for M2 (62.42) was significantly higher than that for M6 (55.32), or M8 (53.08), or M9 (54.96) which indicated the obvious difference in the maintenance of parental methylation situation. The percentage of hypermethylation ranged from 11.7 to 44.9 among the 45 plants and gave an average of 15.98 among the nine lines. The highest value (32.82%) for M8 had no significant differences with those of M6 (29.38%) and M9 (29.14%), but it was significantly higher than M1 (22.84%), M2 (15.98%), M3 (19.90%), M4 (19.96%), M5 (20.76%), and the latter five rates were comparable. The percentage of hypomethylation alterations ranged from 5.50 to 22.70 among the 45 plants and averaged 10.9–19.96 among the lines. The highest percentage for M2 (19.96) was comparable with those for M3 (16.14) and M4 (16.28), but was significantly higher than all the remaining six percentages. The low rates of C type averaged 2.24–5.06 showed no significant differences. The plants from lines M6, M8, and M9 showed higher methylation changes, while those from M2 changed least. This represented that the progenies from different addition lines gave obvious difference in increase or decrease in methylation levels.

**Table 2:** Methylation-sensitive amplified polymorphism analysis of cytosine methylation patterns in the extracted *B*. *napus*.

*Eco*RI Digest (isoschizomer) lane^∗^				Methylation pattern in		Subtype of methylation
*Hpa*II	*Msp*I	*Hpa*II	*Msp*I	*B. napus*	Addition Lines	
–	–	–▾	+▴	#CCGG GGCC	CCGG GGCC	B3
–	–	+	–	CCGG GGCC	CCGG GGCC	B4
–	+	+	+	CCGG GGCC	CCGG GGCC	B1
+	–	+	+	CCGG GGCC	CCGG GGCC	B2
+	+	–	+	CCGG GGCC	CCGG GGCC	A2
+	+	+	–	CCGG GGCC	CCGG GGCC	A1
–	+	–	–	CCGG GGCC	CCGG GGCC	A3
+	–	–	–	CCGG GGCC	CCGG GGCC	A4
–	+	+	–	CCGG GGCC	CCGG GGCC	C1
+	–	–	+	CCGG GGCC	CCGG GGCC	C2
+	+	+	+	CCGG GGCC	CCGG GGCC	D1
+	–	+	–	CCGG GGCC	CCGG GGCC	D2
–	+	–	+	CCGG GGCC	CCGG GGCC	D3

*#The combination of four letter represents the specific restriction endonuclease sequence and the underscored letter represent the restriction endonuclease digestion site. ▴Represented presence (+) of a band resulted from the *Eco*RI/*Hpa*II-digested^∗^ or *Eco*RI/*Msp*I-digested^∗^ genomic DNA of parental *B*. *napus* and nine additions. ▾ Represented absence (-) of a band resulted from the *Eco*RI/*Hpa*II-digested^∗^ or *Eco*RI/*Msp*I -digested^∗^ genomic DNA of parental *B*. *napus* and nine additions. ++,+-, -+ and -- represented CCGG/GGCC or C5^*m*^CGG/GGCC, 5^*m*^CCGG/GGCC, C5^*m*^CGG/GGC5^*m*^C and 5^*m*^CCGG/GGCC5^*m*^, respectively.*

**Table 3:** Methylation-sensitive amplified polymorphism analysis of DNA methylation types in the extracted *B*. *napus*^∗^.

Line	Type A^▴^	Type B^▾^	Type C^■^	Type D^•^
M1	22.84^bc#^	15.20^bc^	4.22^a^	59.68^abc^
M2	15.98^c^	19.96^a^	2.58^a^	62.42^a^
M3	19.90^c^	16.14^ab^	4.04^a^	59.90^ab^
M4	19.60^c^	16.28^ab^	5.06^a^	58.66^abc^
M5	20.76^c^	15.40^bc^	3.30^a^	61.66^ab^
M6	29.38^ab^	11.56^cd^	3.62^a^	55.32^bc^
M7	23.34^bc^	14.62^bcd^	2.24^a^	58.80^abc^
M8	32.82^a^	10.90^d^	3.18^a^	53.08^c^
M9	29.14^ab^	13.40^bcd^	2.48^a^	54.96^bc^
Mean	23.75	14.83	3.41	58.28

*^∗^All values are in percentage. ^#^Values in each column marked with identical letter (s) are not significant (*p* < 0.05). ▴Type A represented hypermethylation. ▾Type B represented hypomethylation. ■Type C represented varied methylation. ^•^Type D represented hemi-methylation or no methylation.*

**FIGURE 3:**
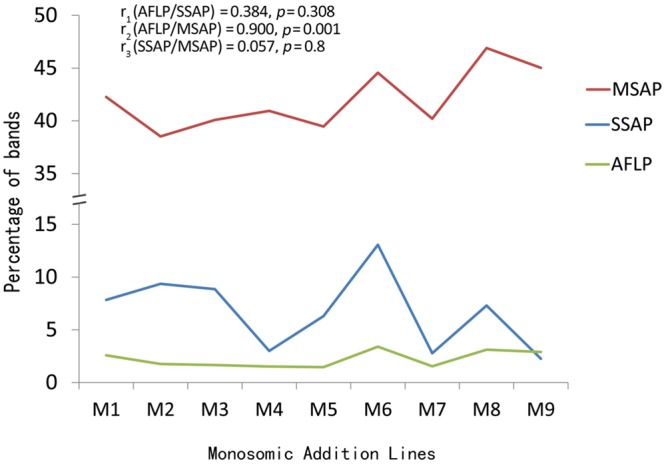
**Correlations of the AFLP, SSAP and MSAP bands among the extracted *B. napus*-type plants**.

The correlation coefficients between AFLP and SSAP was 0.384 (*p* = 0.308), between AFLP and MSAP was 0.900 (*p* = 0.001), and between SSAP and MSAP was 0.057 (*p* = 0.80) (**Figure 3**). Therefore, the AFLP and MSAP values were significantly correlated, AFLP and SSAP changes were some but insignificantly correlated, while SSAP and MSAP changes were not correlated.

## Discussion

Consequent to the merger of the genomes of *B. napus* and *O. violaceus* via somatic hybridization and repeated backcrossing, the genomes of the resultant *B. napus*-type progenies showed changes in the genomic components in terms of (i) losing and acquiring DNA segments and transposons, (ii) introgression of alien DNA segments, and (iii) DNA methylation (**Figures 2** and **3, Tables 1** and **3**), which were likely to be induced during the process of allopolyploidization and aneuploidization and to be maintained, even after the genomes became independent. The variations observed in the three types of the genomic changes named above among the *B. napus*-type individuals extracted from these addition lines (M1–M9) suggested the different effects of individual alien chromosomes from *O. violaceus* on inducing genetic alterations in the *B. napus* genomes. Nevertheless, the change rates obtained here should underestimate the actual changes, because the twice backcrossing of the allohexaploid (2*n* = 62, genomes AACCOO) with parental *B. napus* would diminish the actual alterations responding to the genome merger. The genomic alteration showed no significant correlation with transposon mobilization but found to have a “very strong positive correlation” with DNA methylation changes; no correlation between transposon mobilization and DNA methylation changes. It warrants further investigation to establish a causal link between the two variables showing a “strong positive correlation.”

Of the genomic changes, the extent of loss and gain of the DNA segments could be attributed to the two species involved in hybridization, the type of hybridization (sexual or somatic), and the number of hybridization events (Gaeta et al., 2007; Xiong et al., 2011). The hybridization pathways as well as the genetic and the epigenetic changes in the present materials were compared with the previous *B. napus*-type lines derived from sexual cross between *B. napus* cv. Oro and *O. violaceus* (Zhang et al., 2013). As the separation of parental genomes occurred during the mitotic divisions of the hybrids (Li et al., 1995; Li and Ge, 2007), the somatic cells with the chromosomes of either parental species entered meiosis, resulting in two types of progenies by self-pollination, mostly parental *B. napus* and a few mixoploid hybrids. The mixoploid hybrids that again showed the genome separation were successively selected for mixoploidy up to F_5_ generation. However, the altered chromosome behavior in one F_5_ plant led to the formation of F_6_ plants having variable chromosomal complements. Two F_6_ plants produced further progenies which were advanced to the F_20_ generation; the plants of the F_20_ have the karyotype of *B. napus* but with genomic changes. As a result, the genome of these *B. napus*-type progenies passed through several rounds of genome merger and separation, but no backcrossing was done for developing the progenies. The different crossing approach and pedigrees might have contributed to the greater genomic changes, for their average proportions of loss and gain were 7.2 and 5.4% (Zhang et al., 2013), much higher than observed in the present investigation 1.42 and 0.37% (**Figure 2**, Supplementary Table S1). The larger extent of genomic alteration from the sexual cross might be attributed to the occurrence of partial chromosomal complements in F_6_ progeny and the recovery of the *B. napus-*type in later generations. In contrast, the completeness of the chromosomal complement for *B. napus* was maintained in the pedigree from the allohexaploid backcross for the purpose of MAALs. Interestingly, the introgressions derived from the partial hybrids between *B. napus* and *Capsella bursa-pastoris* (Zhang et al., 2013), although the hybrids were backcrossed twice with the parent *B. napus*, revealed genetic and epigenetic changes similar to those in the hybrids from the sexual cross. Of common was that the introgressions were selected from both types of hybrids carrying partial complements of *B. napus* plus some alien chromosomes or even chromosomal segments; more genetic changes may be envisaged to occur via meiotic recombination during karyotype recovery. Although, the *B. napus*-type plants from the mixoploid hybrids expressed some traits specific for *O. violaceus*, but they had no detectable AFLP bands specific to *O. violaceus*, this was likely attributable to the limited number of primers used and the few sequences introgressed. Additionally, the separation of the genomes from the two species during mitotic divisions of the hybrid cells and subsequent inclusions of the genomes into different cells also decreased the chance for chromosomal exchanges. On the contrary, the sequential formations of the allopolyploid, backcrossing progenies and MAALs should induce introgression of alien DNA segments, although no meiotic homoeologous pairing of chromosomes were observed (Zhao et al., 2008; Ding et al., 2013).

The ‘Genome Shock’ hypothesis (McClintock, 1984) proposes that wide hybridization (including allopolyploidy) is associated with the activity of TEs (transposable elements). Studies on several allopolyploids have suggested that allopolyploidization not always trigger TE mobilization (Madlung et al., 2005; Beaulieu et al., 2009; Parisod et al., 2010a; Petit et al., 2010). Moreover, introduction of small amount of foreign chromatin or DNA has been shown to cause widespread misregulation of TEs, resulting in their mobilization in the genomes of both plants and animals (Remus et al., 1999; Shapiro, 2010). *Athila*-like reterotransposon, a class I TE, is 10 kb long (Alix and Heslop-Harrison, 2004), mainly concentrated in the pericentromeric regions of the chromosome and represented by *c*. 400 copies in *Brassica oleracea* (Alix et al., 2005). Activation of *Athila* retrotransposon was reported for *Arabidopsis* (Josefsson et al., 2006). *Bot1*, a class II CACTA transposon, is also 10 kb long and has *c*. 1500 copies in *B*. *oleracea* and has *Brassica* C-genome specificity and several rounds of amplification, whereas *Brassica* A genome is nearly devoid of *Bot1* copies (Alix et al., 2008). *Bot1* which represented 2.3% of the genome in *B*. *oleracea* played a major role in *Brassica* genome divergence and gene proliferation (Alix et al., 2008). *Brassica* miniature inverted-repeat transposable element (MITE) *BraSto* is 250 bp long, and preferentially located in gene space with *c*. 125 and *c*. 310 copies in *B*. *oleracea* and *B*. *napus*, respectively (Sarilar et al., 2011). Introgression of foreign DNA may lead to the mobilization of MITEs which likely affect gene expression and phenotypic variability (Nakazaki et al., 2003). Therefore, both *Athila*-like reterotransposon and *Bot1* have the same length (10 kb) and are much longer than *BraSto* (250 bp). The average frequency of the SSAP lost bands for these three TEs was rather close (2.18% for *Athila*-like, 2.23% for *Bot1*, 1.80% for *BraSto*) in the *B. napus*-type individuals, while the average of the SSAP new bands for *BraSto* (0.50%) was higher than those of *Athila*-like (0.04%) and for *Bot1* (0) (**Table 1**). Although *Bot1* was distributed on C genome only, it was lost at similar rate as the other two TEs on A and C genome; this likely resulted from the higher copy number of *Bot1*. The lower values of the new bands for *Athila*-like reterotransposon and *Bot1* might be associated with their longer length. In contrast to the SSAP changes of the nine LTR (*Copia*) retrotransposons identified in *B. rapa* in the *B. napus* extracted from the two sexual hybrids mentioned above, the rates were ∼1% lower for both lost and new bands, and the difference was not so obvious as in the AFLP bands. These results from sexual and somatic hybridizations suggested that the genome of *B. napus* responded to TE mobilization within limited ranges, upon genome merger and alien introgression.

DNA methylation perturbation is observed in various allopolyploids or in the F_1_ hybrids (Shaked et al., 2001; Lukens et al., 2006; Gaeta et al., 2007; Hegarty et al., 2011). Extensive and genome-wide alterations in DNA methylation patterns were associated with the integration of alien DNA into the host genome of plants and mammals (Remus et al., 1999; Wang et al., 2005). In the *B. napus*-type progenies from two sexual crosses mentioned above, the rates of methylation changes are similar (33.4–39.8%) and the hypermethylation was more frequent than hypomethylation. Coincidentally, the extent of the total and two degrees of methylation alterations were quite close to those in the present study (**Table 2**); this represented the general trend of methylation in the *B. napus* genome induced by interspecific hybridization and incorporation of alien DNA.

The ND in which the rRNA genes from one parent are expressed has been observed in many interspecific hybrids and their allopolyploid derivatives (Chen and Pikaard, 1997). Furthermore, the co-occurrence of ND with the phenotypic and transcriptomic dominance was shown in certain allopolyploids (Chen, 2007). Based on the suggestion that the ND and the genome-specific stability in natural and synthetic *Brassica* allotetraploids were related, it was proposed that ND possibly played a role in the stabilization of the chromosomes from the parent of the dominant rRNA genes (Ge et al., 2013), given that the rRNAs were the main component of the ribosome for protein synthesis. The rRNA genes from *O. violaceus* were completely dominant over those of *B. napus* in their allohexaploids (Ge et al., 2009). The genes on the *O. violaceus* chromosome in one addition line (M4) expressed complete dominance, while those carried by the alien chromosome in M5 and M6 showed partial dominance (Ding et al., 2013); M6 had the lowest transcription level. It appears that the expression levels of rRNA genes from *O. violaceus* in the three additions namely, M4, M5, and M6 were positively correlated with the genomic stability. The loss rates of ALFP bands in the *B. napus*-type plants from M4 (0.52%) and M5 (0.46%) were lowest among all rates and were significantly much lower than that of M6 (2.92%) (**Figure 2**, Supplementary Table S1). Notably, the rates of lost, new and *O*. *violaceus*-specific bands were very close within plants from M4 or M5, but the rate of loss was much higher than the rates of other two from M6. Similarly, the loss rates of three transposons were lowest in plants from M4 and were not significantly lower than those of M5, but the rates from both M4 and M5 were significantly lower than that of M6 (**Table 1**). The percentage of hypermethylation change in plants from M4 and M5 were quite similar but significantly lower than that of M6, and inversely, the percentage of hypomethylation change in plants from M4 were insignificantly higher than that of M5 but significantly higher than that of M6. The mechanism underlining the relationship between ND and genome stability warrants further investigation.

## Conclusion

The extracted *B. napus* from the intergeneric allopolyploids and additions with another crucifer showed certain alterations in genomic components, retrotransposon mobilization, and DNA methylation. The result firstly indicated the genetic and the epigenetic changes of the genome from one parental species induced by the processes of allopolyploidization and aneuploidization, which were still maintained after the genome was extracted and the parent restituted. The possible effect of the nucleolar dominance on the genomic changes provided novel insights into the genome interaction in the allopolyploids.

## Author Contributions

Conceived and design the experiment: ZL, XG, YS, MG. Performed the experiment: YD, MG. Analyzed the data: ZL, XG, MG, YD. Contributed reagent/material/analysis tool: MG, YD, XG. Write the paper: ZL, MG.

## Conflict of Interest Statement

The authors declare that the research was conducted in the absence of any commercial or financial relationships that could be construed as a potential conflict of interest.

## Abbreviations

AFLP: amplified fragment length polymorphism
MAALs: monosomic alien addition lines
MSAP: methylation-sensitive amplified polymorphism
SSAP: sequence-specific amplified polymorphism
