# Supplementary material for: Genetic and Epigenetic Changes in Oilseed Rape (Brassica napus L.) Extracted from Intergeneric Allopolyploid and Additions with Orychophragmus
Source: Front Plant Sci. 2016 Apr 12;7:438. doi: 10.3389/fpls.2016.00438 (PMC4828432; doi:10.3389/fpls.2016.00438)
Supplement: Supplementary file 1 [file Table_1.DOCX]

**SUPPLEMENTARY TABLE S1 | Statistical analysis of the AFLP bands in the *B. napus*-type plants*****.**

| **Line** | **Lost band** | | **New band** | **Specific band** | **Total** |
| --- | --- | --- | --- | --- | --- |
| M1 | | ^↑A^ 1.90^a#^ | ^B^ 0.30^bc^ | ^B^ 0.38^abc^ | 2.58 |
| M2 | | ^A^ 0.70^b^ | ^A^ 0.54^a^ | ^A^ 0.52^a^ | 1.76 |
| M3 | | ^A^ 0.72^b^ | ^A^ 0.46^a^ | ^A^ 0.48^ab^ | 1.66 |
| M4 | | ^A^ 0.52^b^ | ^A^ 0.50^a^ | ^A^ 0.50^a^ | 1.52 |
| M5 | | ^A^ 0.46^b^ | ^A^ 0.48^a^ | ^A^ 0.52^a^ | 1.46 |
| M6 | | ^A^ 2.92^a^ | ^B^ 0.22^c^ | ^B^ 0.26^c^ | 3.40 |
| M7 | | ^A^ 0.62^b^ | ^B^ 0.40^ab^ | ^AB^ 0.52^a^ | 1.54 |
| M8 | | ^A^ 2.54^a^ | ^B^ 0.24^c^ | ^B^ 0.34^bc^ | 3.12 |
| M9 | | ^A^ 2.40^a^ | ^B^ 0.22^c^ | ^B^ 0.28^c^ | 2.90 |
| Mean | | 1.42 | 0.37 | 0.42 | 2.21 |

^*^All values are percentages.

^↑^Values for a given kind of band across a monosomic line marked with an identical capital letter (s) were not significant (*p*<0.05).

^#^ Values in each column marked with an identical letter (s) were not significant (*p*<0.05).
