# Supplementary material for: Genetic and Epigenetic Changes in Oilseed Rape (Brassica napus L.) Extracted from Intergeneric Allopolyploid and Additions with Orychophragmus
Source: Front Plant Sci. 2016 Apr 12;7:438. doi: 10.3389/fpls.2016.00438 (PMC4828432; doi:10.3389/fpls.2016.00438)
Supplement: Supplementary file 2 [file Table_2.DOC]

**SUPPLEMENTARY TABLE S2** | **List of primer sequences used for amplified fragment length polymorphisms (AFLPs).**

| **Primer name** | **Sequence (5′ to 3′)** |
| --- | --- |
| *Mse*I adapter 1 | GACGATGAGTCCTGA G |
| *Mse*I adapter 2 | TACTCAGGACTCAT |
| *Eco*RI adapter 1 | CTCGTAGACTGCGTACC |
| *Eco*RI adapter 2 | AATTGGTACGCAGTC |
| *Eco*RI pre-selective primer | GACTGCGTACCAATTCA |
| *Mse*I pre-selective primer | GATGAGTCCTGAGTAAC |
| *Eco*RI selective primer 1 | GACTGCGTACCAATTCAAA |
| *Eco*RI selective primer 2 | GACTGCGTACCAATTCAAT |
| *Eco*RI selective primer 3 | GACTGCGTACCAATTCAAC |
| *Eco*RI selective primer 4 | GACTGCGTACCAATTCAAG |
| *Eco*RI selective primer 5 | GACTGCGTACCAATTCATA |
| *Eco*RI selective primer 6 | GACTGCGTACCAATTCATT |
| *Eco*RI selective primer 7 | GACTGCGTACCAATTCATC |
| *Eco*RI selective primer 8 | GACTGCGTACCAATTCATG |
| *Eco*RI selective primer 9 | GACTGCGTACCAATTCACA |
| *Eco*RI selective primer 10 | GACTGCGTACCAATTCACT |
| *Eco*RI selective primer 11 | GACTGCGTACCAATTCACC |
| *Eco*RI selective primer 12 | GACTGCGTACCAATTCACG |
| *Eco*RI selective primer 13 | GACTGCGTACCAATTCAGA |
| *Eco*RI selective primer 14 | GACTGCGTACCAATTCAGT |
| *Eco*RI selective primer 15 | GACTGCGTACCAATTCAGC |
| *Eco*RI selective primer 16 | GACTGCGTACCAATTCAGG |
| *Mse*I selective primer 1 | GATGAGTCCTGAGTAACAA |
| *Mse*I selective primer 2 | GATGAGTCCTGAGTAACAT |
| *Mse*I selective primer 3 | GATGAGTCCTGAGTAACAC |
| *Mse*I selective primer 4 | GATGAGTCCTGAGTAACAG |
| *Mse*I selective primer 5 | GATGAGTCCTGAGTAACTA |
| *Mse*I selective primer 6 | GATGAGTCCTGAGTAACTT |
| *Mse*I selective primer 7 | GATGAGTCCTGAGTAACTC |
| *Mse*I selective primer 8 | GATGAGTCCTGAGTAACTG |
| *Mse*I selective primer 9 | GATGAGTCCTGAGTAACCA |
| *Mse*I selective primer 10 | GATGAGTCCTGAGTAACCT |
| *Mse*I selective primer 11 | GATGAGTCCTGAGTAACCC |
| *Mse*I selective primer 12 | GATGAGTCCTGAGTAACCG |
| *Mse*I selective primer 13 | GATGAGTCCTGAGTAACGA |
| *Mse*I selective primer 14 | GATGAGTCCTGAGTAACGT |
| *Mse*I selective primer 15 | GATGAGTCCTGAGTAACGC |
| *Mse*I selective primer 16 | GATGAGTCCTGAGTAACGG |
