# Supplementary material for: Genetic and Epigenetic Changes in Oilseed Rape (Brassica napus L.) Extracted from Intergeneric Allopolyploid and Additions with Orychophragmus
Source: Front Plant Sci. 2016 Apr 12;7:438. doi: 10.3389/fpls.2016.00438 (PMC4828432; doi:10.3389/fpls.2016.00438)
Supplement: Supplementary file 3 [file Table_3.DOC]

**SUPPLEMENTARY TABLE S3 |** **List of primer sequences and primer pairs used for sequence-specific amplification polymorphisms (SSAPs).**

|  | **Primer name** | **Sequence (5′ to 3′)** |
| --- | --- | --- |
| Common primers | Adaptor *Eco*RI1 | CTCGTAGACTGCGTACC |
|  | Adaptor *Eco*RI | AATTGGTACGCAGTCTAC |
|  | Eco+0 | GACTGCGTACCAATTC |
|  | E33 | GACTGCGTACCAATTCAAG |
|  | E35 | GACTGCGTACCAATTCACA |
|  | E36 | GACTGCGTACCAATTCACC |
|  | E55 | GACTGCGTACCAATTCCGA |
|  | E80 | GACTGCGTACCAATTCTAC |
| *Athila*-SSAP | Athila157/17 | TTCTCAATCAGCTTAAA |
|  | Athila40/18 | GTAGTCATCATCAAATCC |
|  | Athila100/18 | GGCAGAGTTCATAGCACT |
| *BraSto*-SSAP | BrMipreamp | GRAAACTGTTCAAYYAATTAR |
|  | BrMiselect | CAAAACATCAAGTATTATG |
| *Bot1*-SSAP | 3Botpreamp | ATTATTTCCGACGACT |
|  | 3Botselect | TCCTTGCTGTTTTCTTGT |
