# Supplementary material for: Genetic and Epigenetic Changes in Oilseed Rape (Brassica napus L.) Extracted from Intergeneric Allopolyploid and Additions with Orychophragmus
Source: Front Plant Sci. 2016 Apr 12;7:438. doi: 10.3389/fpls.2016.00438 (PMC4828432; doi:10.3389/fpls.2016.00438)
Supplement: Supplementary file 4 [file Table_4.DOC]

**SUPPLEMENTARY TABLE S4 | List of primer sequences used for methylation-sensitive amplification polymorphisms (MSAPs).**

| Primer name | Sequence (5' to 3') |
| --- | --- |
| *Eco*RI adaptor1 | CTCGTAGACTGCGTACC |
| *Eco*RI adaptor2 | AATTGGTACGCAGTCTAC |
| *Hpa*II/*Msp*I adaptor1 | GATCATGAGTCCTGCT |
| *Hpa*II/*Msp*I adaptor2 | CGAGCAGGACTCATGA |
| *Eco*RI pre-selective primer | GACTGCGTACCAATTC |
| *Hpa*II/*Msp*I pre-selective primer | ATCATGAGTCCTGCTCGG |
| *Eco*RI selective primer 1 | GACTGCGTACCAATTCAAC |
| *Eco*RI selective primer 2 | GACTGCGTACCAATTCACG |
| *Eco*RI selective primer 3 | GACTGCGTACCAATTCACT |
| *Eco*RI selective primer 4 | GACTGCGTACCAATTCAGT |
| *Eco*RI selective primer 5 | GACTGCGTACCAATTCAAG |
| *Eco*RI selective primer 6 | GACTGCGTACCAATTCACA |
| *Eco*RI selective primer 7 | GACTGCGTACCAATTCACC |
| *Eco*RI selective primer 8 | GACTGCGTACCAATTCAGC |
| *Hpa*II/*Msp*I selective primer 1 | ATCATGAGTCCTGCTCGGTAA |
| *Hpa*II/*Msp*I selective primer 2 | ATCATGAGTCCTGCTCGGTCC |
| *Hpa*II/*Msp*I selective primer 3 | ATCATGAGTCCTGCTCGGTTC |
| *Hpa*II/*Msp*I selective primer 4 | ATCATGAGTCCTGCTCGGTAC |
| *Hpa*II/*Msp*I selective primer 5 | ATCATGAGTCCTGCTCGGTGC |
| *Hpa*II/*Msp*I selective primer 6 | ATCATGAGTCCTGCTCGGTAG |
| *Hpa*II/*Msp*I selective primer 7 | ATCATGAGTCCTGCTCGGTTG |
| *Hpa*II/*Msp*I selective primer 8 | ATCATGAGTCCTGCTCGGTCA |
